# Cou Cou, flying fish and a whole exome please... lessons learned from genetic testing in Barbados

**DOI:** 10.11604/pamj.2021.38.111.27969

**Published:** 2021-02-03

**Authors:** Morris Hilary Scantlebury, Karlene Tanechia Barrett, Simeona Jacinto, David Orlando Christopher Corbin, Marina Kerr, Aneal Khan

**Affiliations:** 1 Departments of Pediatrics and Clinical Neuroscience, Cumming School of Medicine, University of Calgary, Calgary, Alberta, Canada,; 2 Queen Elizabeth Hospital, Bridgetown, Barbados,; 3 Discovery DNA Incorporated, Calgary, Alberta, Canada,; 4 Department of Medical Genetics, Cumming School of Medicine, University of Calgary, Calgary, Alberta, Canada

**Keywords:** Neurogenetic disorders, epilepsy, Caribbean

## Abstract

Millions of patients, with suspected complex neurogenetic disorders, living in resource limited regions around the world have no access to genetic testing despite the rapidly expanding availability and decreasing costs of genetic testing in first world nations. The barriers to increasing availability of genetic testing in resource limited nations are multifactorial but can be attributed, in large part, to a lack of awareness of the power of genetic testing to lead to a rapid, cost-effective, diagnosis that potentially will have profound clinical implications on treatment and patient outcomes. We report our experience with whole exome sequencing (WES) done for the first time in 5 patients of African descent with a suspected neurogenetic disorder living in a resource limited setting on the Eastern Caribbean island of Barbados. A diagnostic pathogenic mutation was found in 3 patients in the SCN1A, STXBP1 and SCN4A, who clinically were diagnosed with Dravet syndrome, Lennox-Gastaut syndrome, paramytonia and seizures respectively. A variant of undetermined significance was found in a patient with global developmental delays, hypotonia, with abnormal eye movements. In one patient WES was non-diagnostic. This result highlights the high yield of WES in carefully selected patients with a neurologic disease and the need for increase access to genetic testing in resource limited settings globally.

## Commentary

Barbados is a small (431 km^2^) island located in the Eastern Caribbean with a population of 287,375 and is known for its warm weather, beautiful beaches and friendly people. Its national dish is Cou Cou (cornmeal served with okra, a staple dish for Africans brought to America during slavery) and flying fish [1]. The public health care system is delivered through a network of polyclinics scattered throughout the island, with tertiary services provided through the Queen Elizabeth Hospital located in the capital city, Bridgetown. There are 2 adult neurologists and only one functions in the public setting (DOCC). A visiting pediatric neurologist (MHS) has been conducting sessional, conjoint, adult and pediatric neurology clinics, mainly in the public sector for the past eight years. At the multidisciplinary government-run Child Development Center, undiagnosed cases of chronic epileptic encephalopathies and intractable epilepsy are common and place a major burden on the health care system. Regrettably, genetic testing is unavailable as it is for millions of patients living with undiagnosed neurogenetic disorders in resource limited regions (RLR), despite the rapidly expanding availability and decreasing costs of genetic testing in developed nations [2]. We report our experience with whole exome sequencing (WES) performed for the first time in 5 patients of African ancestry, with an undiagnosed neurogenetic disorder living in Barbados. A genetic diagnosis was obtained in 4 out of 5 cases with a dual diagnosis in one case (Table 1). This result highlights the high yield of WES in carefully selected patients with an undiagnosed neurogenetic disorder and the need to increase access to genetic testing in RLR globally.

**Table 1:** summary of the clinical features and diagnostic exome sequencing results

	Age	Clinical	Gene	Changes Reported
Case 1	23 months	Dravet syndrome	SCN1A, NM_001165963.1	c.3662A>C, p.Glu1221Ala
Case 2	15 months	Hypotonia, developmental delay and abnormal eye movements	SPTBN2, NM_006946.2	c.5057G>A, p.Arg1686Gln
Case 3	19 years	Intractable epilepsy since infancy and severe intellectual disability	CNNM2, NM_017649.4	c.14G>T, p.Gly5Val
Case 4	25 years	Paramyotonia congenita and a remote history of seizures	SCN4A, NM_000334.4	c.3938C>T,p.Thr1313Met
			IDS, NM_000202.7	c.596A>G, p.Lys199Arg
Case 5	11 years	Lennox-Gastaut syndrome	STXBP1, NM_003165.3	c.874C>T, p.Arg292Cys

, in developed nations, has seen an explosion in the clinical availability and impact of genetic testing since sequencing of the entire human genome was completed in 2003 [3]. The recent advances in next generation sequencing (NGS) technology to sequence thousands of genes simultaneously, combined with the added portability for remote collection of DNA samples using saliva kits, allows patients in RLR to access services without the need to invest in expensive laboratories locally. As costs for NGS testing continue to decrease, it is becoming increasingly reasonable and affordable to use NGS as a first line diagnostic test. Furthermore, using saliva kits allows for non-invasive sample collection that doesn´t require the expertise of a medical professional and as the collected sample is stored/collected in preservative, it is stable for up to 5 years at room temperature. This allows for non-expedited, room temperature shipping, and thus reduced shipping costs. WES, which sequences the coding regions of genes (exons), covers only about 1.5% of the genome but accounts for more than 80% of known human single gene diseases, thus presenting a cost-effective approach to making a diagnosis in RLR. In fact, all samples in this project were gifted through Discovery DNA (Calgary, Alberta), and using a high throughput targeted DNA capture approach, allowed for additional samples to be uniquely indexed and added to an existing exome sequencing batch without compromising sequencing output, but reducing cost per sample to less than US$750. This approach expands the ability to facilitate testing for RLR at a fraction of the cost.

Importantly, WES has unleashed a new world of possibilities for the management of patients with undiagnosed neurogenetic disorders. The discovery of novel treatments for neurogenetic disorders is happening at an unprecedented pace, due to advances in high-throughput drug screening in pre-clinical neurogenetic models, coupled with simplification of gene editing processes. High throughput drug screening has revolutionized attempts to identify treatments through repurposing of approved drugs, which may profoundly affect the management of neurogenetic disorders in RLR as newly discovered treatments may already be widely available and highly affordable. However, the obvious first step to improving the lives of patients with complex neurogenetic disorders living in RLR is to increase access to genetic testing.

What then are the barriers to increasing genetic testing in RLR? In our opinion the barriers are context dependent and multifactorial, but can be attributed, in large part, to a lack of awareness of the capabilities of NGS for a rapid, cost-effective approach that can have clinical implications on treatment and patient outcomes for a broad range of single gene disorders. Examples of an effect on treatment are even evident in our small case series. Take into consideration the patient with a SCN1A mutation, sodium channel blockers will be avoided as this can make seizures worst. Efforts to rule out abnormal potassium levels during an acute attack of paralysis will be made in the patient with the SCN4A mutation along with a metabolic work-up for IDS, although there is no clinical evidence for Hunter syndrome, as these are treatable. A search for hypomagnesemia will be made in the patient with the variant in *CNNM2*, as this too is treatable. WES was confidently concluded with clinical correlation diagnostic for patients with *SCN1A, SCN4A, STXBP1*, thus avoiding additional costly tests, exposure to dangerous ineffectual treatments and expensive far away trips to international institutions seeking a diagnosis. Long-term management strategies informed by the genetic findings can be instituted early, and with the genetic diagnosis in hand, physicians and patients can be on the look-out for affordable, potential new treatments as they arise. However, to carefully select patients for genetic testing, specialist consultations are required even if remotely.

Some may argue that the cost to implement WES more broadly is still too high, even without the need for major investments in infrastructure. Do we then wait until the cost falls further before we act? Of course not! The World Health Organization has already embraced the challenge and created the human genomics and global health initiative that aims to raise awareness, facilitate access and promote the development of cost-effective and safe quality health services in RLR arising from the application of genomics. In the Americas, the Pan American Health Organization (PAHO) has adopted resolution CD55.R12: access and rational use of strategic and high-cost medicines and other health technologies which recognizes that improving equitable access to and the rational use of medicines and other health technologies contributes to achieving universal access to health and universal health coverage and the achievement of sustainable development goals. Resolution CD55.R12 is supported by the PAHO-Strategic Fund (SF) which is an innovative cooperative funding model with a mission to improve access to high-quality medicines and health technologies in the Americas including the Caribbean. Use of the PAHO-SF has grown yearly since 2005 and in 2016 alone the PAHO-SF has granted 17 lines of credit amounting to a total of ~$9M USD. Although access to genetic testing is competing with other priorities such as malnutrition and HIV, note that for rare diseases which affects ~470 million people worldwide, neurogenetic is the most common etiology. Therefore, genetic testing remains a major unmet need that urgently requires addressing [4, 5].

Our hope is that genetic testing becomes readily affordable as Cou Cou, and like flying fish (which can spread their large pectoral fins and travel great distances) is able to reach people everywhere living with undiagnosed neurogenetic disorders as it is currently being achieved in developed nations.
